# Vitamin D3 Ameliorates DNA Damage Caused by Developmental Exposure to Endocrine Disruptors in the Uterine Myometrial Stem Cells of Eker Rats

**DOI:** 10.3390/cells9061459

**Published:** 2020-06-12

**Authors:** Hoda Elkafas, Mohamed Ali, Engy Elmorsy, Rehab Kamel, Winston E. Thompson, Osama Badary, Ayman Al-Hendy, Qiwei Yang

**Affiliations:** 1 Department of Pharmacology and Toxicology, National Organization for Drug Control and Research (NODCAR), Cairo 35521, Egypt; helkaf2@uic.edu; 2 Department of Surgery, University of Illinois at Chicago, Chicago, IL 60612, USA; mali78@uic.edu (M.A.); aalhendy@uic.edu (A.A.-H.); 3 Department of Clinical Pharmacy, Faculty of Pharmacy, Ain Shams University, Cairo 11591, Egypt; 4 Department of Pharmacology and Toxicology, Faculty of Pharmacy, Helwan University, Cairo 11795, Egypt; elmorsy_em@yahoo.com (E.E.); kamelrehab@yahoo.com (R.K.); 5 Department of Physiology, Reproductive Science Research Program, Morehouse School of Medicine, Atlanta, GA 30310, USA; wthompson@msm.edu; 6 Department of Clinical Pharmacy, Faculty of Pharmacy, British University in Egypt, Cairo 11837, Egypt; Obadary@yahoo.com

**Keywords:** uterine fibroids, myometrial stem cells, endocrine-disrupting chemicals, DNA damage, vitamin D3

## Abstract

Early-life exposure of the myometrium to endocrine-disrupting chemicals (EDCs) has been shown to increase the risk of uterine fibroid (UF) prevalence in adulthood. Vitamin D3 (VitD3) is an unique, natural compound that may reduce the risk of developing UFs. However, little is known about the role and molecular mechanism of VitD3 on exposed myometrial stem cells (MMSCs). We investigated the role and molecular mechanism underlying VitD3 action on DNA damage response (DDR) defects in rat MMSCs due to developmental exposure to diethylstilbestrol (DES), with the additional goal of understanding how VitD3 decreases the incidence of UFs later in life. Female newborn Eker rats were exposed to DES or a vehicle early in life; they were then sacrificed at 5 months of age (pro-fibroid stage) and subjected to myometrial Stro1+/CD44+ stem cell isolation. Several techniques were performed to determine the effect of VitD3 treatment on the DNA repair pathway in DES-exposed MMSCs (DES-MMSCs). Results showed that there was a significantly reduced expression of RAD50 and MRE11, key DNA repair proteins in DES-exposed myometrial tissues, compared to vehicle (VEH)-exposed tissues (*p* < 0.01). VitD3 treatment significantly decreased the DNA damage levels in DES-MMSCs. Concomitantly, the levels of key DNA damage repair members, including the MRN complex, increased in DES-MMSCs following treatment with VitD3 (*p* < 0.01). VitD3 acts on DNA repair via the MRN complex/ATM axis, restores the DNA repair signaling network, and enhances DDR. This study demonstrates, for the first time, that VitD3 treatment attenuated the DNA damage load in MMSCs exposed to DES and classic DNA damage inducers. Moreover, VitD3 targets primed MMSCs, suggesting a novel therapeutic approach for the prevention of UF development.

## 1. Introduction

Uterine fibroids (UFs) are the most common benign monoclonal tumors of the reproductive tract. They originate from myometrial stem cells and serve as one of the most frequent reasons for myomectomies and hysterectomies [1,2]. Although benign, UFs can cause profound gynecologic and reproduction conditions ranging from prolonged bleeding, anemia, and pelvic pain to recurrent abortion, subfertility, and preterm labor [3]. These complications impart an economic burden, and it is estimated that upwards of $34.4 billion is spent annually in the United States alone on treating the condition and the complications arising from it [4]. Currently, there are no FDA approved drugs used for the long-term treatment of UFs. Therefore, surgery remains the standard treatment, with the condition negatively affecting the quality of life for women and especially affecting women of color, who experience a higher prevalence of UFs [5].

Environmental exposures that contribute to increasing UF pathogenesis have been reported [6]. There is accumulated evidence suggesting that exposure to hormones during development may be associated with a risk for UFs [7,8]. Endocrine-disrupting chemicals (EDCs) can be either synthesized or natural hormones; these are substances in our environment, food, and consumer products that interfere with hormone biosynthesis, metabolism, and action, resulting in a deviation from normal homeostatic control or reproduction [9,10,11]. Developmental exposure to EDCs such as diethylstilbestrol (DES), phthalates, and soy phytoestrogen genistein contributes to the increase in the incidence (and size) of UFs in animal models [5,12]. Epidemiologic studies have also detected a correlation between an increased risk for early UF diagnosis and in utero DES exposure, with the use of soy-based formulas in infancy also being implicated as a risk factor [13,14,15]. In 1971, DES was the first pharmaceutical estrogen to be orally administrated to reduce the risk of spontaneous abortion or preterm labor in pregnant women. It is now implicated in vaginal tumors [16,17].

There is a shortage of data needed to identify the mechanisms through which environmental EDCs may alter the human epigenome; thus, it is challenging to provide clear, clinical directions when discussing the implied health effects of unavoidable, low-level exposures that are typical in populations. Additionally, due to ethical concerns, human studies addressing environmental exposures are limited. Accordingly, animal models afford the opportunity to investigate possible connections between early-life exposure to EDCs and related disorders [5]. Eker rats, which carry a germ-line mutation in the tuberous sclerosis complex 2 (*Tsc2*) tumor suppressor gene, are susceptible to UFs and provide a suitable animal model for studying gene-environment interactions, especially as DES exposure increases the *Tsc2* penetrance, tumor multiplicity and size [18,19]. We previously isolated cells from healthy rat myometria using Stro1/CD44 surface markers, which showed properties of myometrial stem/progenitor-like cells (MMSCs). These Stro1+/CD44+ MMSCs also reacted to environmental signals—contracting with age and expanding in response to developmental and environmental exposures that promote UF pathogenesis. In humans, the number of MMSCs in normal myometria was found to be correlated with the risk of developing UFs. An elevated number of MMSCs were observed in Caucasian women with UFs in comparison to those without UFs. African American women, the group with the highest risk for these tumors, exhibited the highest number of MMSCs. Taken together, these findings indicate that Stro1+/CD44+ MMSCs as myometrial stem/progenitor cells are markers for environmental factors that contribute to the increased risk for UFs [20].

Defects in the DNA damage response (DDR) network are implicated in the development of a variety of diseases and cancer-predisposition syndromes [21]. In DDR genes, accumulated mutations in the DNA may result in malignancies. In UFs, early-life exposure to EDCs such as DES, not only alters the expression of genes and phosphorylation of proteins required for DNA repair, but also may permanently modify the main functionality of DNA repair capacity of the MMSCs by inhibiting their ability to repair DNA double-strand breaks (DSBs) and driving an increased tumor predilection in adulthood [5]. In human, 70% of UFs exhibit somatic mutations in *MED12*, and *MED12* mutations have been observed only in UF stem cells, representing the primary difference between MMSCs and UF stem cells. [22], Furthermore, genetic instabilities, including chromosomal loss and rearrangement, have also been observed in UFs. These studies suggest that dysfunctions in the DNA repair pathway may be a driving force behind tumor progression [7,23,24,25].

It is well known that 1,25-dihydroxyvitamin D3 (VitD3) plays an important role in calcium homeostasis and control of bone metabolism. The hormonal structure of VitD3 has a powerful anticancer effect [26,27], including a protective effect on colorectal cancer as well as prostate and breast cancer [28]. We have previously demonstrated that VitD3 has an inhibitory effect on the UF phenotype and decreases the size of the UFs [29,30,31]. It may also diminish the accumulation of DNA damage in UF tissues [32]. Earlier studies have demonstrated that low serum levels of VitD3 and downregulated vitamin D receptor expression have been observed with UFs, with increases in DNA damage and decreases in DNA repair factors, including the double-strand break (DSB) sensors, the MRN complex, mediators, and effectors in UF cells [33,34,35]. In addition, a study by Granziano et al. [36] showed that knockdown of vitamin D receptor in human fibroblasts downregulated the expression of homologous recombination (HR)-related repair genes, including RAD51 and BRCA1, and increased γ-H2AX levels. Another study using an animal model showed that diet-induced vitamin D deficiencies triggered DNA damage in mouse myometrial tissue [37,38]. Therefore, in the present study, we assessed whether VitD3 treatment of MMSCs from Eker rats with early-life exposure to DES will reverse the EDC-induced DNA damage by increasing DNA repair capacity. The aim of this study is to provide further insights on the preventive role of VitD3 in protecting against UF development later in women’s lives.

## 2. Materials and Methods

### 2.1. Uterine Fibroid Animal Model

The Eker rat model was used to study UF pathogenesis. Female (Long Evans; *Tsc2* Ek/+) newborn Eker rats were obtained from an on-site colony and subcutaneously injected with 10 µg of DES (Sigma-Aldrich, St. Louis, MO, USA) per rat per day (*n* = 5) or with 50 µL of sesame seed oil (vehicle (VEH), *n* = 5) on days 10, 11, and 12 after birth (PND 10–12), a critical period for uterine development. According to the protocol described previously, the animals were maintained and sacrificed at the age of 5 months (representing the early adult stage) [19]. The animal model and experimental design are shown in Figure S1. Protocols involving the use of these animals were approved by the Committee on the Ethics of Animal Experiments, University of Illinois at Chicago (protocol #18040).

### 2.2. Isolation of Eker Rat MMSCs, Cell Culture, and Treatment

Tissue digestion and stem cell isolation were performed as described previously [8]. Briefly, uterine tissues from DES (*n* = 5) and VEH (*n* = 5) Eker rats were collected and washed 3 times in freshly prepared buffer solution. Endometrial and serosal tissues were removed from myometrial tissues by scraping with an antiseptic scalpel to ensure that only myometrial tissue was used for stem cell isolation. The tissues were digested and prepared for single-cell suspensions. The cells were then subjected to MMSCs isolation using Stro1/CD44 dual surface markers by fluorescence-assisted cell sorting [8]. Isolated DES- and VEH-exposed MMSCs (VEH-MMSCs) (1 × 10^6^) were seeded in a collagen-coated T-75 culture flask vent cap. The cells were grown in SmBM growth media (Lonza, Walkersville, MD, USA) supplemented with 5% fetal bovine serum, 0.1% insulin, 0.2% recombinant human fibroblast growth factor, 0.1% gentamicin sulfate and amphotericin B mixture, and 0.1% human epidermal growth factor and kept under hypoxic conditions (37 °C, 5% CO_2_, 2% O_2_). Once they reached 60% confluence, DES-MMSCs were treated with VitD3 (100 nM) for 48 h. Absolute ethanol was used as a vehicle to dissolve the drugs. The final concentration of ethanol in the culture media was <0.01%. The cells were then trypsinized, washed with PBS, and centrifuged at 1500 rpm for 5 min. The supernatant was discarded, and the pellets were snap-frozen in liquid nitrogen and stored at −80 °C for subsequent use.

### 2.3. Reagents and Antibodies

Bioactive VitD3 and Mirin (MRN-ATM pathway inhibitor) were purchased from Sigma Biochemicals (St Louis, MO, USA). Camptothecin (CPT) was purchased from Abcam (Cambridge, MA, USA). SmBM was purchased from Lonza (Walkersville, MD, USA). Antibodies used in this study are shown in Table 1.

### 2.4. Immunohistochemistry (IHC) Analysis

Myometrial tissues were fixed in 10% buffered formalin for 15–20 h, then embedded with paraffin and subjected to IHC. Briefly, paraffin-embedded, myometrial tissue sections from DES- and VEH-exposed animals were made into single-layer tissue slides for staining and analysis with classic IHC. Staining and imaging were performed by the Research Histology and Tissue Imaging Core at the University of Illinois at Chicago. For quantitative analysis, the protein immunostaining positive intensity was measured using Aperio image scope analysis software (Aperio Technologies, Inc., Vista, VA, USA).

### 2.5. Western Blot Analysis

Cell pellets were lysed in RIPA buffer with a protease and phosphatase inhibitor cocktail (Thermo Fisher Scientific, Waltham, MA, USA). Protein quantification was carried out using the Bradford method (Bio-Rad Protein Assay kit, Hercules, CA, USA), where 30 µg protein lysate from different samples was resolved in Gradient (4–20%) Mini-PROTEAN TGX Precast Protein Gels (Bio-Rad, Hercules, CA, USA) and transferred to a polyvinylidene fluoride membrane (Bio-Rad). The membrane was blocked at room temperature for 1 h in either 5% *w*/*v* nonfat dry milk or 5% bovine serum albumin in 0.05% Tween-supplemented PBS. The membrane was then incubated overnight with the specific primary antibodies listed in Table 1 at 4 °C before a 90-min incubation with horseradish peroxidase conjugated secondary antibodies (Cell Signaling Technology, Danvers, MA, USA). The antigen–antibody complex was detected with the Pierce enhanced chemiluminescence detection kit (Trident Femto Western HRP Substrate (GeneTex, Irvine, CA, USA). Specific protein bands were visualized using the ChemiDoc XRS+ molecular imager (Bio-Rad, Hercules, CA, USA) [32,39]. Quantification of each protein band’s intensity was undertaken using Bio-Rad Image Lab software and normalized against the corresponding β-actin.2.6. Immunofluorescence and Laser Confocal Microscopy

DES/VEH-MMSCs (2 × 10^4^) were cultured on Nunc Lab-Tek II-CC2 4-chamber slides. After reaching 60% confluence, the cells were treated with VitD3 at 100 and 500 nM for 48 h. Ethanol was used as a vehicle. Cells were fixed using BD Cytofix/Cytoperm Fixation/Permeabilization kit (BD Biosciences, San Jose, CA, USA) at room temperature for 15 min. After rinsing with PBS 3 times, the cells were permeabilized for 15 min, and nonspecific binding was blocked using a blocking/incubation solution containing 1% BSA in 0.1% Triton X-100/PBS for 1 h. Cells were incubated overnight with primary anti-γ-H2AX (1:200), anti- P95/NBS-1antibody (1:100.) at 4 °C followed by incubation with anti-rabbit Alexa Fluor 488-conjugated secondary antibody (Cell Signaling Technology, Danvers, MA, USA) (1:1000 each) for 1 h at room temperature. Cells were washed for 15 min (3 washes, 5 min for each) with the above-described incubation solution, air-dried, and mounted on microscopic slides with one drop of Fluorshield (Sigma) containing 4′,6-diamidino-2-phenylindole for nuclear staining [5]. Fluorescent signals were imaged using a Zeiss laser scanning confocal microscope and 710 META ZEN Black 2012 confocal software. Images were captured at 63× magnification using a 63× Plan-Apo (oil), 1.4 NA lens and exported using Zen Blue software (Zeiss AG, Oberkochen, Germany).

### 2.6. Flow Cytometry Analysis

MMSCs were stained for DNA repair and damage markers using intracellular staining after fixation and permeabilization with a Fix/Perm kit (BD Biosciences, San Jose, CA, USA). The intracellular staining for proteins was done in the presence of GolgiStop (BD Biosciences). A total of 1 × 10^5^ cells were incubated with selected antibodies for 30 min at room temperature [38]. Data were analyzed using FACSCanto (BD Biosciences) and FlowJo systems (Ashland, OR, USA).

### 2.7. RNA Isolation and Quantitative Real-Time PCR

Quantitative real-time PCR was performed to determine the mRNA expression of various genes using primer sequences listed in Table 2. Primers were purchased from Integrated DNA Technologies (IDT, Coralville, IA, USA). The same volume of cDNA from each sample was added to the master mix, including proper primer sets and SYBR green super mix (Bio-Rad) in a 20 µL reaction volume. All samples were analyzed in triplicate. Real-time PCR analyses were performed using Bio-Rad CFX96. Cycling conditions, including denaturation, were performed at 95 °C for 2 min, followed by 40 cycles of 95 °C for 5 s and 60 °C for 30 s, and then at 65 °C for 5 s. A melting-curve analysis affirmed the synthesis of a DNA product of the predicted size. The expression data were normalized using 18S ribosomal RNA values (housekeeping gene), and these normalized values were used to generate data graphs. A reaction without a cDNA template was used as a negative control.

### 2.8. Cell Proliferation (MTT) Assay

Cells were seeded in a 96-well, flat-bottom microliter plate at a density of 5000 cells/well and left to adhere for 24 h at 37 °C in a hypoxia incubator. Cells were then treated with VitD3 (100 nM) for 48 h. The MTT solution (with a working concentration of 5 mg/mL PBS) was added to each well, and the plate was incubated for 4 h at 37 °C in a CO_2_ incubator. The medium was then aspirated, and the formed formazan crystals were solubilized by adding 100 μL of DMSO per well for 30 min at 37 °C in a CO_2_ incubator. The intensity of the dissolved formazan crystals (purple color) was quantified using an ELISA plate reader at 540 nm.

### 2.9. Statistical Analyses

For quantitative RT-PCR data, gene expression results are displayed as the fold change ± standard error of the mean (SEM). Bio-Rad CFX Manager 3.1 software was used for the statistical analysis. Experiments were performed in triplicate. For the Western blot analysis, results are presented as the mean ± standard deviation (SD) and analyzed using the two-tailed unpaired Student’s *t*-test. Experiments were performed in triplicate and repeated twice. For the immunofluorescence and IHC analysis, the signal intensity was measured as the median ± 95% confidence interval and analyzed using the nonparametric Mann–Whitney test. Variances were estimated to be statistically significant at * *p* < 0.05, ** *p* < 0.01, and *** *p* < 0.001. GraphPad 5.0 (La Jolla, CA, USA) was used to create the graphs.

## 3. Results

### 3.1. Developmental DES Exposure Increased DNA Damage by Altering the DNA Repair Pathway in MMSCs

**Figure 1:**
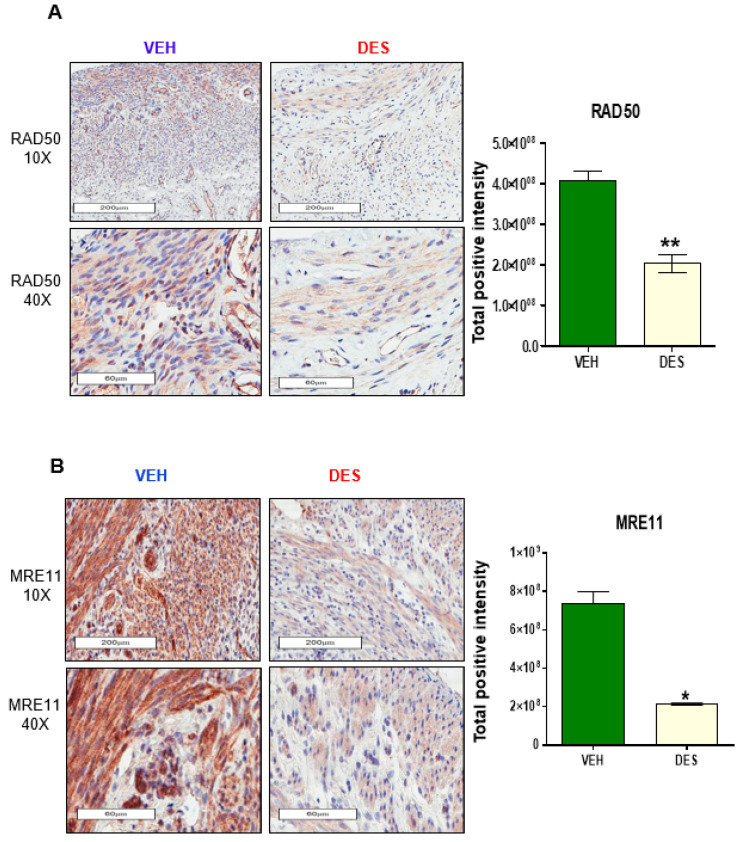
Early-life exposure to diethylstilbestrol (DES) attenuates the expression of RAD50 and MRE11 in rat myometria. (**A**) IHC staining of DNA repair protein RAD50 in myometria of 5-month-old Eker rats at postnatal day (PND) 10–12 exposed to DES and vehicle (VEH) (left panel), and quantitative analysis of RAD50 positive nuclei in DES-exposed vs. VEH-exposed rat myometrial tissue (right panel). (**B**) IHC staining of DNA repair protein MRE11 from the myometria of 5-month-old Eker rats at PND10-12 exposed to DES and VEH (left panel), and quantitative analysis of MRE11 positive nuclei in DES-exposed vs. VEH-exposed rat myometrial tissue (right panel). The percentage of positive cells with brown nuclei was calculated based on the number of stained cells, counted in a total of five random 20× power fields in five DES-exposed Eker rat myometrial tissues and VEH-exposed myometrial tissues (* *p* < 0.05 ** *p* < 0.01).

To determine if developmental exposure to DES altered the DSB repair pathways in the myometrium, the protein levels of the DNA repair sensor proteins MRE11 and RAD50 were evaluated using IHC. As shown in Figure 1A (left and right panels), RAD50 positive cells were significantly decreased in DES-exposed compared to VEH-exposed myometria (** *p* < 0.001). Similarly, the positive cells of MRE11, another key MRN complex member, were also significantly decreased in DES-exposed myometria compared to VEH-exposed myometria (Figure 1B, * *p* < 0.01).

### 3.2. VitD3 Attenuated DES-Exposure-Induced DNA Damage in MMSCs and Upregulated the Expression of Protein Phosphokinases

**Figure 2:**
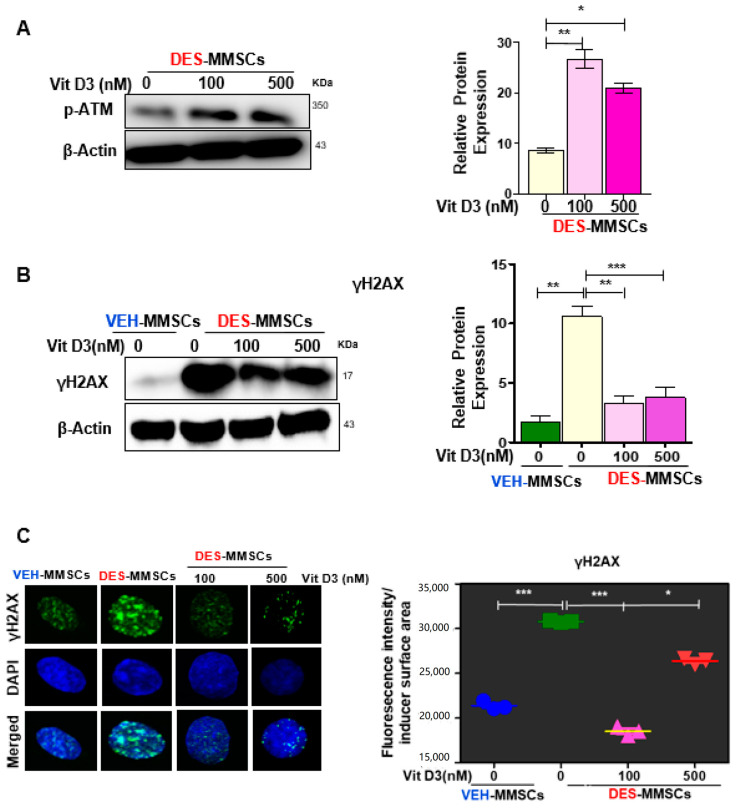
VitD3 reverses the impairment of DNA repair capacity in DES-exposed myometrial stem cells (DES-MMSCs). (**A**) The left panel shows the Western blot of p-ATM in DES-MMSCS with and without VitD3 (48 h), where β-actin was used as a loading control. The right panel shows the corresponding data graph created using relative data. (**B**) The protein levels of the DNA damage marker H2AX in MMSCs were determined by Western blot analysis. VitD3 (48 h treatment) protected DES-MMSCs from double-strand breaks (DSBs) and promoted DSB DNA repair (left panel). Relative data were used to create the corresponding data graph (right panel). (**C**) The left panel shows immunofluorescence staining of the γH2AX antibody in VEH- and DES-MMSCs with and without VitD3 treatment using confocal laser microscopy. The right panel shows single γ-H2AX foci altered fluorescence intensity per nuclear area assessed by Image J. Quantification of nuclear γH2AX intensity was used to compare DES-MMSCs with VEH-exposed MMSCs (VEH-MMSCs) and to compare VitD3 treatment at 100 and 500 nM with untreated DES-MMSCs. Two-tailed unpaired Student’s *t*-tests were used. Experiments were performed in triplicate and repeated twice with *n* = 3 biological replicates. All graphs show mean ± SEM with * *p* < 0.05, ** *p* < 0.01, *** *p* <0.001.

MMSCs are the cellular origin of UFs and exhibit accumulated DNA damage following early-life exposure to EDCs, and our previous study showed that the ratio of p-ATM to ATM protein expression was lower in DES-exposed MMSCs (DES-MMSCs) as compared to VEH-MMSCs [5]. To determine whether VitD3 treatment is able to restore the activity of ataxia-telangiectasia mutated (ATM) kinase in DES-MMSCs, p-ATM levels were measured by Western blot analysis. As shown in Figure 2A, a significant increase in response to 48 h VitD3 treatment was observed at concentrations of 100 and 500 nM (* *p* < 0.5 and ** *p* < 0.001, respectively). Then, we determined whether VitD3 treatment was capable of reducing DNA damage in DES-MMSCs. As shown in Figure 2B, we first confirmed that DES-MMSCs accumulated more DNA damage than VEH-MMSCs, as indicated via γH2AX protein expression. VitD3 was capable of reversing the DNA damage sensor γH2AX in MMSCs caused by developmental DES exposure. Western blot analysis demonstrated that the treatment of DES-MMSCs with VitD3 (100 and 500 nM) for 48 h significantly decreased the levels of γH2AX (** *p* < 0.01 and *** *p* < 0.001, respectively). In addition, immunofluorescence analysis confirmed the same findings that VitD3 treatment at 100 and 500 nM significantly decreased the number of γH2AX foci in MMSCs (*** *p* < 0.001 and * *p* < 0.05, respectively) as shown in Figure 2C.

The MRN complex, consisting of MRE11, RAD50, and NBS1, is responsible for the initial processing of DSBs before DNA repair and decreased expression of DNA repair genes is correlated with a reduction in DNA repair capacity. To determine whether the downregulation of MRN proteins was due to the deactivation of transcription levels in DES-MMSCs or VitD3 is capable of restoring their gene expression levels, the qRT-PCR analysis was performed to measure the RNA levels of several genes related to DNA DSB repair, including MRE11, RAD50, NBS-1, BRCA1, BRCA2, RAD51, Chk1, Chk2, and ATM. As shown in Figure S2A, a significant downregulation of the all these mentioned genes in DES-MMSCs was observed compared to VEH-exposed MMSCs (* *p* < 0.05, ** *p* < 0.01, *** *p* < 0.001). Moreover, the treatment of DES-MMSCs with VitD3 was able to restore the gene expression of all of the measured DNA repair genes.

**Figure 3:**
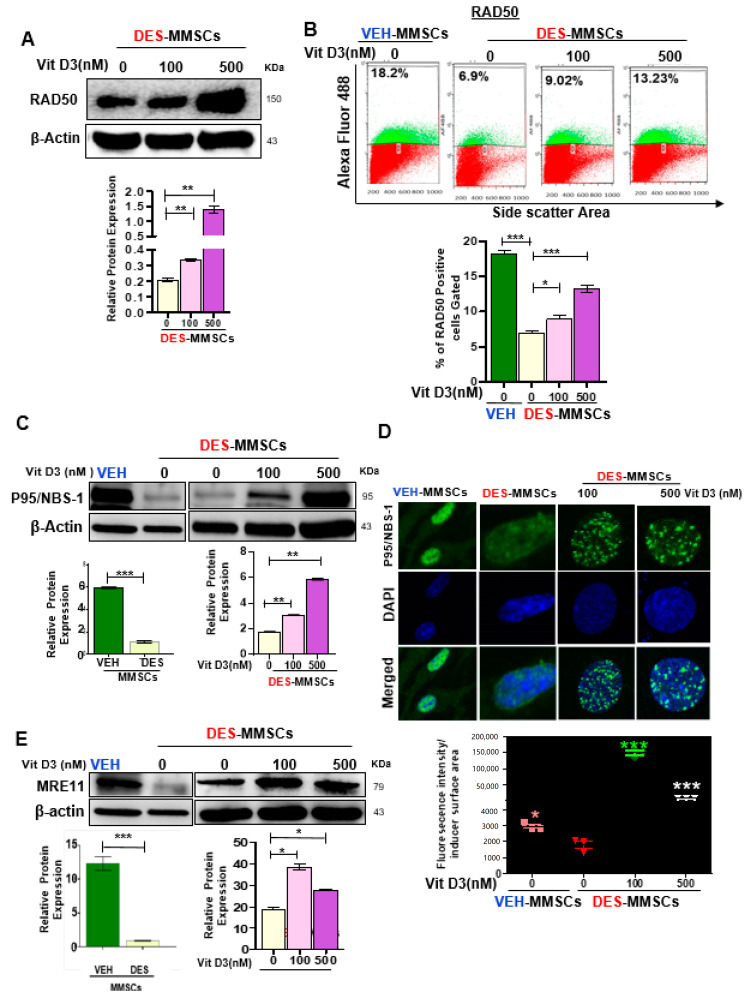
VitD3 increases the expression of DNA repair sensors (RAD50, p95/NBS-1, and MRE11) in DES-MMSCs. (**A**) The protein levels of RAD50 were determined by Western blot analysis (top panel). A quantitative analysis of Western blot data was performed by Image lab software (bottom panel). (**B**) Flow cytometry analysis of RAD50 was performed in the presence or absence of VitD3 (100 nM) (top panel). Data are represented as mean fluorescence intensity after intracellular staining of RAD50 in DES-MMSCs from Eker rats at PND10–12 exposed to DES (bottom panel). (**C**) The protein levels of p95/NBS-1 were determined by Western blot in untreated VEH-MMSCs as well as DES-MMSCs in presence or absence of VitD3 treatment. (**D**) Immunofluorescence staining of P95/NBS-1was performed in untreated VEH-MMSCs as well as DES-MMSCs in the presence or absence of VitD3 treatment. P95/NBS-1foci reflecting the DNA DSB sensor were assessed using immunofluorescence staining and confocal laser microscopy (63× imaging) (top panel). Individual data points are P95/NBS-1foci corrected fluorescence intensity per nuclear area measured by ImageJ. Data are shown as the mean ± SEM (bottom panel) (**E**). Western blot analysis was performed to determine the protein levels of MRE11 in untreated VEH-MMSCs as well as DES-MMSCs in the presence or absence of VitD3 treatment (top panel). The intensity of each band was quantified and normalized to the corresponding β-actin. Relative values were used to generate the data graph (bottom panel) as mean ± SD with * *p* < 0.05, ** *p* < 0.01. *** *p* < 0.001.

To characterize the functional link between the MRN complex and DNA damage in response to VitD3 treatments, we measured the RAD50 protein levels by Western blot in DES-MMSCs in response to VitD3 treatment, and our data showed a significant increase in protein levels of RAD50 (Figure 3A, *p* < 0.01). Moreover, flow cytometry analysis was performed to measure the intracellular staining of nuclear RAD50. As shown in Figure 3B, RAD50 protein levels were significantly decreased in DES-MMSCs as compared to VEH-MMSCs (*** *p* < 0.001). Treatment of DES-MMSCs with VitD3 at 100 and 500 nM resulted in an increased expression of RAD50 (* *p* < 0.05, and *** *p* < 0.001, respectively), confirming the WB results. Moreover, as shown in Figure 3C, VitD3 treatment significantly increased the protein levels of NBS1 in MMSCs (*** *p* < 0.001). To confirm the increase in the expression of NBS1 in DES-MMSCs treated with VitD3 immunofluorescence staining demonstrated that VitD3 was capable of increasing the number of NBS1 foci in MMSCs (Figure 3D, *** *p* < 0.001). MRE11, one of the MRN members, also showed upregulation of protein levels in response to VitD3 treatment (100 and 500 nM) in MMSCs (Figure 3E, *** *p* < 0.001). In addition, we confirmed the significantly lower expression of NBS1 and MRE11 in DES-MMSCs at protein levels compared to VEH-MMSCs (Figure 3D,E respectively), in accordance with the RNA levels in Figure S2A.

To confirm the WB results, we next determined whether VitD3 treatment was capable of enhancing the levels of MRN gene expression in DES-MMSCs. As shown in Figure S2B, VitD3 treatment at both 100 and 500 nM significantly increased the RNA expression of MRE11 and NBS1. While VitD3 at 100 nM did not affect the expression of RAD50, at 500 nM, it did increase the gene expression of RAD50. These results suggest that transcription activation may contribute to the increased protein levels of MRN members in response to VitD3 treatment (* *p* < 0.05, ** *p* < 0.01). Moreover, to determine the effect of VitD3 on the RNA expression of DNA repair mediators, q-PCR was performed to measure the RNA levels in DES-MMSCs treated with VitD3. As shown in Figure S2C, VitD3 treatment at both low (100 nM) and high doses (500 nM) showed upregulation of the BRCA1 expression in DES-MMSCs. Furthermore, a significant upregulation of Chk1 and Chk2 was also observed in DES-MMSCs treated with 500 nM of VitD3 (** *p* < 0.01 and *** *p* < 0.001, respectively).

**Figure 4:**
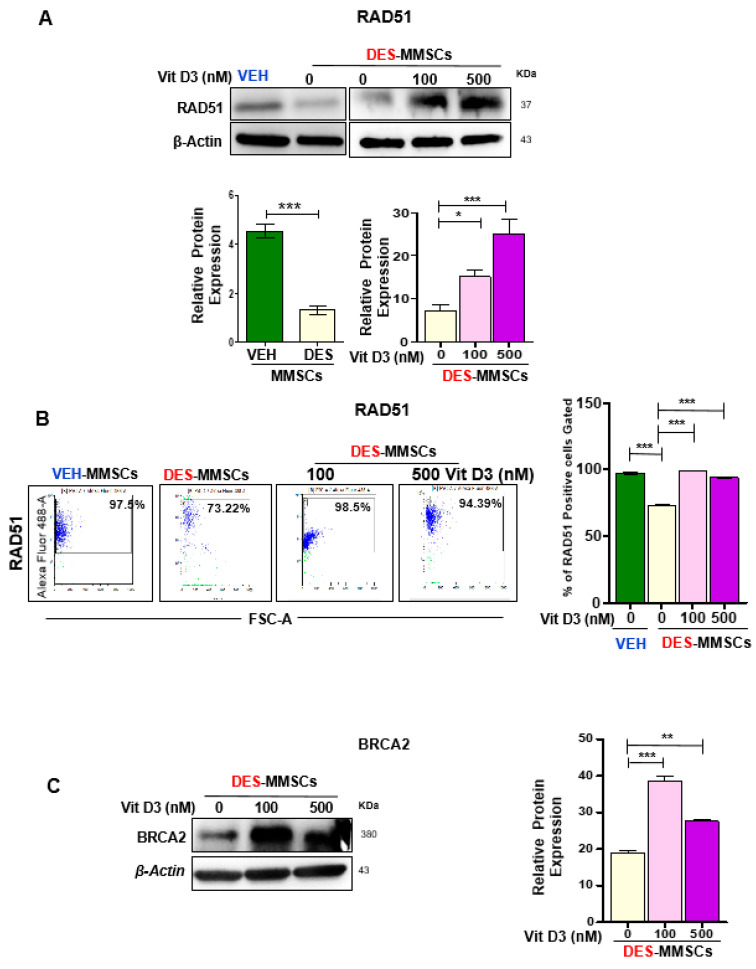
The effect of VitD3 treatment on the expression of DNA repair effectors in DES-MMSCs. Protein lysates were prepared from untreated VEH-MMSCs as well as DES-MMSCs in the presence or absence of VitD3 treatment. (**A**) Western blot analysis was performed to determine the protein levels of RAD51 (top panel). The intensity of each band was quantified and normalized to the corresponding β-actin; relative values were used to generate the data graph as mean ± SD (bottom panel). (**B**) Flow cytometry analysis of RAD51 was performed in untreated VEH-MMSCs as well as DES-MMSCs in the presence or absence of VitD3 treatment (left panel). Data are represented as mean fluorescence intensity after intracellular staining of RAD51 (right panel). (**C**) Western blots measured the protein levels of BRCA2 after the treatment of DES-MMSCs with VitD3 (left panel). Quantitative analysis of BRAC2 was performed using Image J (right panel). Mean ± SD values were used to build the data graph, with * *p* < 0.05, ** *p* < 0.01, and *** *p* < 0.001.

The DSB effector RAD51 forms a helical nucleoprotein filament on DNA and interacts with single-strand DNA (ssDNA)-binding proteins such as BRCA2 to play a role in DNA damage repair. Therefore, we measured the RAD51 and BRCA2 expression levels in MMSCs in the presence or absence of VitD3. As shown in Figure 4A, first, we demonstrated that DES-MMSCs expressed significantly lower levels of RAD51 protein compared to VEH-MMSCs (*** *p* < 0.001). Interestingly, VitD3 treatment increased the protein levels of RAD51 in a dose-dependent manner (*** *p* < 0.001). Flow cytometry analysis further demonstrated that RAD51 expression was downregulated in DES-MMSCs as compared to VEH-MMSCs, while treatment with VitD3 increased the RAD51 expression in DES-MMSCs (Figure 4B). Moreover, treatment of DES-MMSCs with VitD3 significantly upregulated BRCA2 protein levels in DES-MMSCs (** *p* < 0.01 and *** *p* < 0.001) (Figure 4C). The RNA expression levels of both RAD51 and BRCA2 were significantly increased in response to VitD3 treatment at 500 nM in DES-MMSCs (** *p* < 0.01, *** *p* < 0.001), suggesting that increases in RAD51 and BRCA2 expression with VitD3 treatment are partially due to an increase in transcriptional activity (Figure S2C).

### 3.3. VitD3 Attenuated Mirin- and CPT-Induced DNA Damage in DES-MMSCs

**Figure 5:**
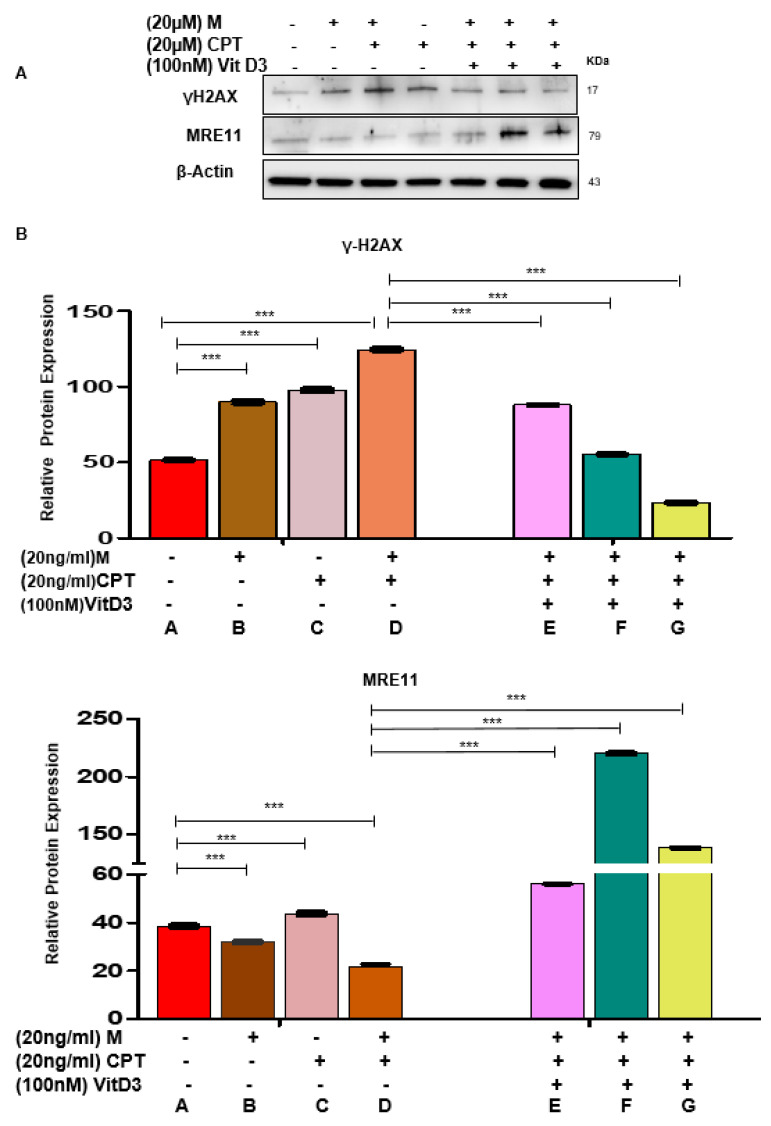
VitD3 antagonizes Camptothecin (CPT)- and Mirin-induced DNA damage in DES-MMSCs. (**A**) The protein levels of the DNA damage sensor γH2AX and MRE11 were measured in DES-MMSCs with DNA-damage-inducer agents Mirin and CPT (20 µg/mL) individually and in combination. The last three lanes represent the effect of VitD3 (100 nM) on the γH2AX and MRE11 levels in DES-MMSCs treated with CPT in combination with Mirin at different time points (1, 9, and 12 h). (**B**) Relevant data were used to create the corresponding data graph for γH2AX (top panel) and MRE11 (bottom panel) in DES-MMSCs treated with VitD3 at different time points (1, 9, and 12 h) with *** *p* < 0.001. Full experimental design and explanation of different groups (A–G) are shown in Figure S3.

**Figure 6:**
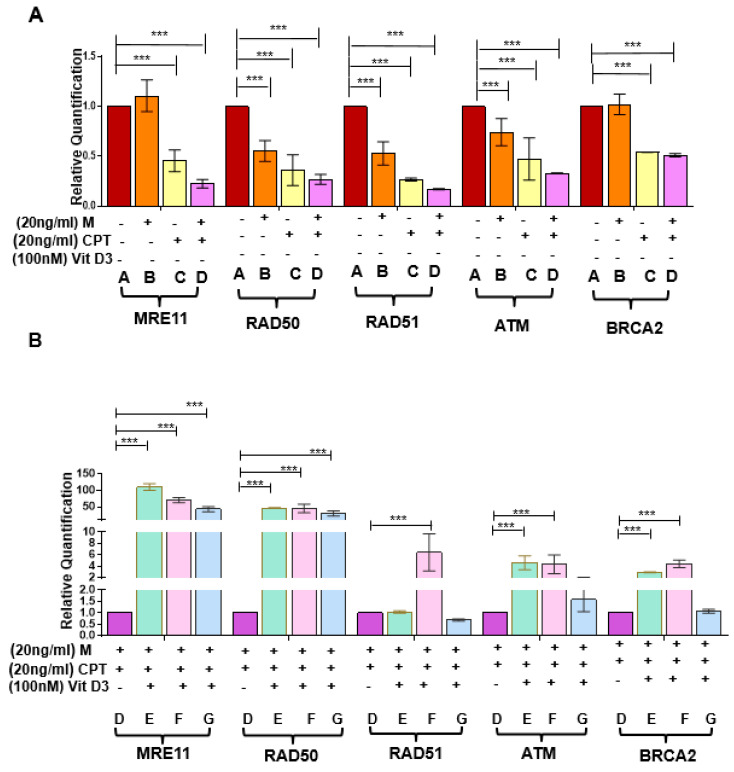
Effect of VitD3 on RNA expression of DNA repair genes in the presence of DNA damage inducers. (**A**) mRNA expression of DSB repair sensors (MRE11 and RAD50), effectors (BRCA2, RAD51), and ATM in DES-MMSCs treated with the double-strand DNA damaging agents CPT and Mirin. (**B**) The mRNA expression levels of DSB repair sensors (MRE11 and RAD50), effectors (BRCA2, RAD51), and ATM were measured in Mirin- and CPT-treated DES-MMSCs with and without VitD3 at different time points. Full experimental design and explanation of different groups (A–G) can be found in Figure S3. The mRNA levels were normalized to 18S rRNA, and normalized values were used to generate the graph. Data are presented as mean ± SEM of triplicate measurements with *** *p* < 0.001.

To further determine the role of VitD3 in reversing the DNA damage in MMSCs, two DNA damage inducers (Mirin and CPT) were used to induce DNA damage. Mirin is an extensively validated pharmacological inhibitor of MRE11 exonuclease activity, and CPT is a selective inhibitor of topoisomerase I, which causes DNA damage. Based on the literature, Mirin induces DNA damage in 9 h and CPT in 3 h [40,41], so our experimental design was to expose DES-MMSCs to either Mirin for 9 h or CPT for 3 h or together with or without VitD3 treatment (one group was to expose the cells to VitD3 for 1 h before adding the DNA damage inducers), making the overall experimental time points 1, 9 and 12 h. A detailed illustration of the experimental design and different treatment groups for results shown in Figure 5 and Figure 6 and Figure S3. As shown in Figure 5A, these DNA-damage-inducing agents significantly increased the levels of γH2AX by approximately 1.6- (group B) and 2-fold (group C), respectively. In addition, the combination treatment exhibited even more DNA DSBs, showing a 2.5-fold increase of γH2AX levels (group D) in DES-MMSCs compared with control group A (*** *p* < 0.001). Concomitantly, protein levels of MRE11 were decreased by 1.2-fold after Mirin treatment. The combination treatment showed downregulation in the MRE11 protein by 2-fold, suggesting that MRE11 and topoisomerase activities may play a critical role in maintaining genomic stability in MMSCs.

Next, we determined whether VitD3 treatment could reverse the adverse effects caused by these two DNA damage inducers. As shown in Figure 5A,B VitD3 treatment reduced the Mirin + CPT induced DNA DSBs in a time-dependent manner (*** *p* < 0.001). Western blot analysis demonstrated that VitD3 treatment was capable of decreasing the γH2AX protein levels in Mirin + CPT treated DES-MMSCs by approximately 1.5-fold (group E). However, increasing the duration of VitD3 with Mirin for 9 h showed a downregulation of γH2AX levels by approximately 2.3-fold (group F) compared to the combination treatment (group D). An increase in the treatment time of VitD3 in DES-MMSCs with DNA damage exhibited a greater reduction of γH2AX (group G), by approximately 6-fold (Figure 5B, top panel).

We then investigated the effects of Mirin and CPT treatments on the levels of MRE11 in DES-MMSCs. As shown in Figure 5A,B a single treatment with Mirin or CPT changed MRE11 protein levels significantly. However, combination treatment showed the lowest decrease in the protein levels of MRE11 (group D). VitD3 treatment increased the reduced levels of MRE11 with the CPT and Mirin combination treatment by 2.5-fold (group E). An increase in the VitD3 treatment time to 9 h showed upregulation of MRE11 by 10-fold (group F) compared to DES-MMSCs treated with CPT and Mirin in the absence of VitD3 (group D). Moreover, VitD3 treatment for 12 h also increased MRE11 protein levels by approximately 6.2-fold (group G) in the presence of Mirin and CPT (Figure 5A,B, *** *p* < 0.001).

To investigate if Mirin and CPT increased DNA DSBs via downregulating DNA repair gene expression, RNA expression levels were measured in DES-MMSCs treated with Mirin, CPT, or the two in combination. As shown in Figure 6A, the RNA expression levels of RAD50 and RAD51 were downregulated in response to Mirin treatment, but this was not the case for MRE11, ATM, or BRCA2. CPT treatment resulted in a decreased RNA expression of all five measured genes, as mentioned earlier. Moreover, the combination treatment caused further decreases in the RNA expression of MRE11, RAD50, RAD51, and ATM in DES-MMSCs (*** *p* < 0.001).

We determined whether VitD3 treatment was capable of reversing decreased expression levels of DNA damage repair genes induced by Mirin and CPT. As shown in Figure 6B, VitD3 significantly increased the RNA expression of MRE11 and RAD50 at 1, 9, and 12 h of treatment (*p* < 0.01). The reverse effect of VitD3 on ATM and BRCA2 expression in DES-MMSCs treated with Mirin + CPT was also observed at 1 and 9 h of treatment (*p* < 0.001). Moreover, VitD3 treatment for 9 h was able to reverse the reduced expression of RAD51 in MMSCs treated with Mirin + CPT (*** *p* < 0.001).

Finally, we estimated the impact of VitD3 on DES-MMSC cell growth where cells were treated with different concentrations of VitD3 for 48 h, and proliferation was determined by MTT assay (Figure S4). DES-MMSCs showed a higher proliferation rate than VEH-MMSCs (*p* < 0.001), while VitD3 decreased the growth of DES-MMSCs in dose-dependent manner as compared to untreated control (Figure S4). At 48 h treatment, VitD3 significantly reduced the proliferation of prefibrotic stem cells by 1.3-fold at a 100 nM concentration and 1.5-fold at a 500 nM concentration, compared with untreated controls (** *p* < 0.01, *** *p* < 0.001). These studies suggest that VitD3 reduces the proliferation of DES-MMSCs.

## 4. Discussion

This study describes, for the first time, the role of VitD3 in reversing DNA DSBs in MMSCs primed by developmental exposure to DES. Experimental animal studies have previously shown that early-life exposure to DES leads to loss of function of the normal *Tsc2* allele, particularly in the reproductive system, which may develop neoplastic diseases such as UFs. The Eker rat model is a valuable tool for studying gene–environment interactions in tumorigenesis [5,18,42]. Other studies have shown that neonatal exposure to EDCs such as bisphenol A produces significant DNA damage, which induces various carcinogenic diseases later in life [5,43].

Following our study design, we were able to identify alterations in DNA repair capacity in MMSCs isolated from 5-month-old rats (representing the early adulthood period) in response to developmental DES exposure, allowing us to explain the genetic attributes behind the conversion of normal healthy MMSCs to tumor-initiating stem cells. DSBs are the most common type of DNA damage inflicted on the genome by genotoxic exposures such as ionizing radiation or chemicals such as EDCs. Reports in the literature have shown that DNA damage in rapidly proliferating cells accumulates due to the inadequate expression of DNA repair genes and can increase the risk of several diseases, including cancer [44,45]. In a previous study of ours, we showed that the repair capacity of DNA DSBs in DES-MMSCs was significantly reduced compared to VEH-MMSCs. This may cause genomic instability (including loss of the normal *Tsc2* allele) and increase the appearance of UFs in DES-MMSCs [5]. Although loss of *Tsc2* function due to EDC exposure is rare in humans, the DES-induced genetic instability in rat MMSCs mirrors the defect of DNA repair in human MMSCs causing the *MED12* mutation and other genetic instabilities. In this study, we used the animal model to understand the gene–environment interaction that causes pathogenesis of UFs in humans.

A considerable number of DNA repair proteins act on DNA damage to eliminate mutations and other changes. In this study, we investigated whether VitD3 treatment restores the suppressed expression levels of DNA repair proteins in DES-MMSCs caused by early-life exposure to DES. Our research indicates that early-life exposure to DES in Eker rat myometrial tissue decreased the protein levels of RAD50 and MRE11, which are both critical Homologous recombination (HR)-DNA repair sensors. MMSCs isolated from DES-exposed tissues exhibited an elevation in DNA damage levels, with a downregulation of DNA repair proteins crucial for DNA DSB repair. This abnormal decrease in DNA repair protein levels may diminish the functional potential for precisely repairing DNA DSBs. Importantly, we characterized VitD3 action on MMSCs, which are the cellular origins of UFs [5,8,46,47]. A dramatic decrease of γH2AX levels (a DNA damage indicator) in DES-MMSCs showed that VitD3 treatment decreased DNA damage induced by DES exposure. These findings are consistent with a previous study showing that VitD3 represses tumorigenesis by inhibiting oxidative stress and inducing tumor cellular senescence in mice [48,49,50]. In addition, previous studies, by both our group and others, have demonstrated that VitD3 treatment attenuates DNA damage in human UF cells [32,49]. Our current study suggests that VitD3 not only targets the DNA repair pathway in differentiated UF cells, but also plays an essential role in DNA repair in pro-fibroid stem cells.

The MRE11–RAD50–NBS-1 (MRN) complex has emerged as a crucial actor in DSB repair. The MRE11 protein adheres to DNA, maintains both exonuclease and endonuclease activities, and produces the scaffolding between NBS1 and RAD50. RAD50 dimerizes and uses its extended coiled-coil branches to keep the ends of the broken DNA together. The NBS1 protein itself has no enzymatic activity but interferes with many protein–protein interactions that improve the functioning of the MRN complex [51,52]. The abnormal levels of the MRN complex are associated with an increased risk of UFs and the UF phenotype and other carcinogenic diseases such as colorectal cancer [5,30,53,54,55]. To determine the role that the MRN complex plays in VitD3-enhanced DNA repair capacity in primed MMSCs, we measured the levels of RAD50, NBS1, and MRE11 in DES-MMSCs in the presence and absence of VitD3. Our data showed that the three members of the MRN complex exhibited an increase in RNA and protein levels in response to VitD3 treatment. These results suggest that VitD3 improved DNA repair capacity, which is essential for the initiation and activation of the DDR in MMSCs. Our data are also consistent with other studies showing the chemoprotective effect of VitD3 in human cancer cells [56].

A functional MRN complex is necessary for a proper ATM-mediated response to DSBs. Immediately after the DDR pathway is initiated, activation of ATM occurs in connection to the MRN complex. ATM phosphorylates several key proteins that initiate the activation of DNA repair checkpoints. γ-H2AX is an ATM target that acts as a biomarker for DSB [48,57,58,59,60,61]. Considering the importance of ATM activation in DNA repair, we investigated the effect of VitD3 on ATM activation. Our results showed that 100 and 500 nM of VitD3 treatment induced phosphorylation of ATM at ser1981 and promoted DSB repair through the MRN/ATM axis. Previous studies have demonstrated that VitD3, acting as a chemopreventive agent, could be explained by the ATM–vitamin D receptor signal loop [57]. Our current findings are also consistent with a previously published study [60] showing that VitD3 diminished the level of H2AX phosphorylation activation following the induction of DNA damage in a mouse model system.

**Figure 7:**
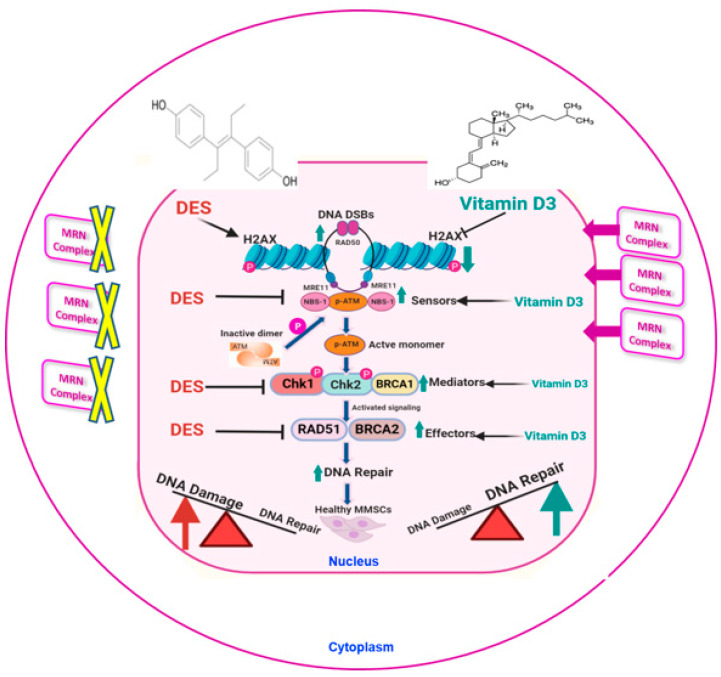
Working model of the relationship between DES-induced DNA damage and the effect of VitD3. Developmental exposure to DES enhances DNA damage load, as demonstrated by increased levels of the DNA damage marker γ-H2AX, and is concomitant with decreases in protein levels of key DNA repair components, including the MRN complex (degraded in the cytoplasm), mediators, and effectors. VitD3 treatment is capable of reversing the action of DES by recovering DNA damage repair capacity through increasing the translocation of the nuclear protein levels of DNA repair proteins, including RAD50, MRE11, and NBS1, as well as effectors and mediators including Chk1, Chk2, BRCA2, and RAD51, thus enhancing the DNA damage repair response. This figure was created with BioRender (https://biorender.com).

ATM-regulated, downstream signaling networks that respond to DNA damage were also investigated. DNA damage repair initiation through the ATM and ATR kinases regulates HR. BRCA1 deficiency contributes to genetic instability and tumorigenesis. Interplays between Chk1 and Rad51 are needed for the function of BRCA1 in Chk1 activation and signaling in response to S-phase and cell-cycle checkpoint regulation [62,63]. Chk1 phosphorylates the recombinase Rad51 and promotes repair of the breakthrough HR through the formation of nucleofilaments mediated by BRCA2 [5,32,64,65,66]. RAD51, as a central recombination enzyme, has also been implicated in replication fork processing. In this regard, RAD51 upregulation by VitD3 treatment may be involved in the replication fork regression in MMSCs [67]. Notably, a recent study demonstrated that BRCA2 interacts with RNase H2, mediates its localization to DSBs, and controls DNA:RNA hybrid levels at DSBs [68], suggesting that VitD3-induced upregulation of BRAC*2* may contribute to HR-mediated repair. Our findings showed that treatment with VitD3 upregulated protein levels of several mediators and effectors, including Chk1, Chk2, BRCA1, RAD51, and BRCA2 in DES-MMSCs (working model shown in Figure 7).

We observed that DES-MMSCs are extremely sensitive to the growth-inhibitory effect of VitD3. Our observation that VitD3 significantly decreased the proliferation of DES-MMSCs suggests a potential role for VitD3 in reducing tumor development (Figure S4). Our current findings are also in agreement with previously published data [69,70].

To fully understand the antitumor activity of VitD3 via the DNA repair pathway in MMSCs, we used chemical compounds that cause the inhibition of DNA repair protein activity and therefore lead to genomic instability. We treated DES-MMSCs with Mirin and CPT, both well-known DNA damage inducers, and investigated whether VitD3 treatment would reverse their effects. Our study demonstrated that VitD3 treatment attenuated the DNA damage induced by Mirin or CPT individually, as well as the damage induced by a combination treatment. Notably, Mirin has been shown to function as an MRE11 inhibitor, inhibiting ATM and the exonuclease activity of MRE11, hence inhibiting the HR pathway [71]. CPT mainly inhibits eukaryotic DNA topoisomerase I by trapping a covalent enzyme-DNA intermediate, CPT-induced single-strand DNA breaks are differentially involved in HR repair by Chk1 and Chk2 [72]. We also observed the different effects of VitD3 in terms of timing treatment. 100 nM VitD3 treatment in DES-MMSCs decreased the γ-H2AX levels by 3.19-fold with a concomitant increase in protein levels of MRE11 by 2-fold, while VitD3 decreased the CPT + Mirin induced γ-H2AX levels by 3.8-fold with a concomitant increase in expression of MRE11 by 6.3-fold. These studies suggest that simultaneous administration of VitD3 with DNA damage inducers or treatment of VitD3 at an early stage of DNA damage may exhibit a more efficient effect of VitD3 on DNA repair.

Our research provides direct evidence that VitD3 treatment suppressed Mirin/CPT-induced DNA damage, suggesting that VitD3 targets the MRN/ATM axis. In addition, we observed that the levels of MRE11 were upregulated in response to VitD3 treatment in MMSCs treated with DNA damage inducers. Based on the animal model used in this study, VitD3 treatment may play a crucial role in preventing UF risk by reshaping the DNA repair signaling network towards increasing the DNA repair capacity and DDR (working model shown in Figure 7).

## 5. Conclusions

This study demonstrates, for the first time, that VitD3 is capable of attenuating DNA damage in MMSCs either by developmental exposure to DES or classic DNA damage inducers. VitD3 acts on DNA repair via the MRN complex/ATM axis, restores the DNA repair signaling network, and enhances DDR. These data suggest that VitD3 treatment may be useful in reversing the action of EDCs and decreasing the incidence of UF development through the amelioration of pathogenic DNA damage. Future VitD3 intervention studies in vivo will afford an opportunity to understand better the effect of VitD3 on reversing the defect of DNA damage repair.

## Figures and Tables

**Table 1:** Antibodies used in the study.

Antibody	Manufacturer	Catalog Number	Species, Monoclonal or Polyclonal	Application and Dilution
γH2AX	Cell Signaling Technology	9718	Rabbit, monoclonal	WB 1:1000 IF and F 1:200
RAD51	Abcam	AB133534	Rabbit, monoclonal	WB 1:5000 F 1:1000
RAD50	Abcam	AB89	Mouse, monoclonal	WB 1:5000
NBS1	Cell Signaling Technology	14959	Rabbit, monoclonal	WB 1:1000 F 1:400
ATM	Abcam	Ab78	Mouse, monoclonal	WB 1:1000
p-ATM	Abcam	AB36810	Mouse, monoclonal	WB 1:1000 F 1µg for 10^6^ cells
BRCA2	Bioss Antibodies	Bs-1210R	Rabbit, polyclonal	WB 1:100–1000
Anti-β actin	Sigma Biochemicals,	A5316	Mouse, monoclonal	WB 1:5000
Alexa Fluor 488 Conjugate	Cell Signaling Technology	4408	Anti-Mouse IgG (H + L), F(ab’)2 Fragment	IF and F 1:500–1:2000
Alexa Fluor 488 Conjugate	Cell Signaling Technology	4412	Anti-Rabbit IgG (H + L), F(ab’)2 Fragment	IF and F 1:500–1:2000

IF: Immunofluorescence, WB: Western blot, F: Flow cytometry.

**Table 2:** Rat primer sequences for RT-qPCR.

Gene Symbol	Gene Description	Forward Primer Sequence	Reverse Primer Sequence
Chk1	Chk1 checkpoint homolog	GGATGAGAAGATACTGGTTGACTT	TCGCTGAGCTTCCCTTTAATC
Chk2	Chk2 checkpoint homolog	GAATTGTTTGACCGTGTGGTG	GTCCCGATGTATGATCCCATTT
ATM	Ataxia Telangiectasia mutated	GCAGTCATCATGCAGACCTATC	GTATCAGAGAACCGCGCTAATG
MRE11	MRE11meiotic recombination 11	TTCTTAAGGAGCGCCACATC	CCTCTCGAACTTCATCGTCTTC
RAD50	RAD50	GCTCGGATATGCTCTTGATG	GAACTGGAGAAGCTGAGTAAAG
NBS1	Nijmegen breakage syndrome protein 1	CAGAAGAGGAAGTGTTGGAAGAG	CATCCATCCTGGGCTTCTTT
RAD51	RAD51	TGCCCTTTACAGAACAGACTAC	ACCACTGCTACACCAAACTC
BRCA1	Breast cancer 1, early onset	GAATCCTGTAAGCCAGAATCCA	CCTTCTCATTCCTGACTCCTTATC
BRCA2	Breast cancer 2, early onset	GCAGATGGTGGATGGCTAAT	TTAGAGACCCAGACGCTAGAA
18S	18S Ribosomal RNA	CACGGACAGGATTGACAGATT	GAGTCTCGTTCGTTATCGGAATTA

## Supplementary Materials

The following are available online at https://www.mdpi.com/2073-4409/9/6/1459/s1, Figure S1: The animal model and experimental design, Figure S2: RNA expressions of DNA repair genes in VEH-MMSCs and DES-MMSCs with or without VitD3. Figure S3: Experimental design of VitD3 effect on DNA damage repair in DES-MMSACs with or without classic DNA damage inducers. Figure S4: The effect of VitD3 on DES-MMSCs proliferation.
