# Supplementary material for: Vitamin D3 Ameliorates DNA Damage Caused by Developmental Exposure to Endocrine Disruptors in the Uterine Myometrial Stem Cells of Eker Rats
Source: Cells. 2020 Jun 12;9(6):1459. doi: 10.3390/cells9061459 (PMC7349254; doi:10.3390/cells9061459)
Supplement: Supplementary file 1 [file cells-09-01459-s001.pdf]

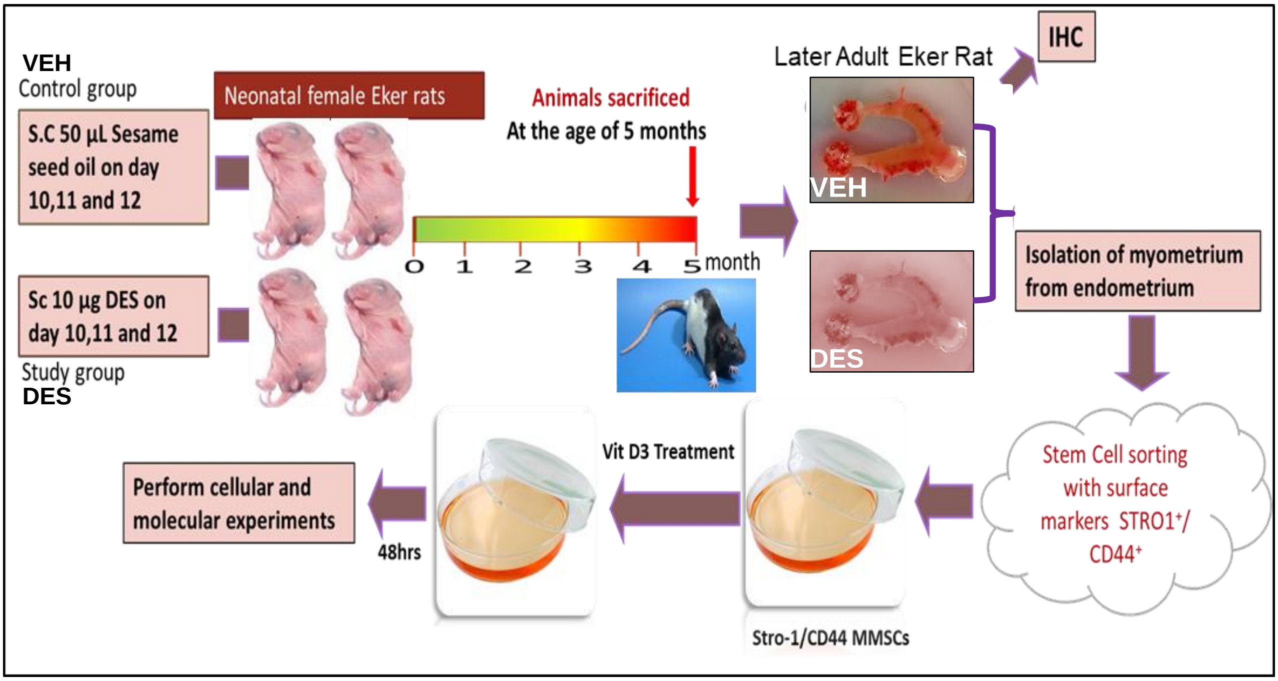

Supplemental Figure 1. In vivo Study design. Eker rat pups were exposed to VEH and DES at postnatal days (10-12), a sensitive period of uterine development. The pups were sacrificed at 5 months of age, which represents the adult stage. Rat myometrial tissue was isolated and subjected to MMSC isolation using Stro-1/CD44 dual surface markers. Cellular and molecular experiments were performed using myometria and MMSCs from both DES- and VEH-exposed Eker rats.

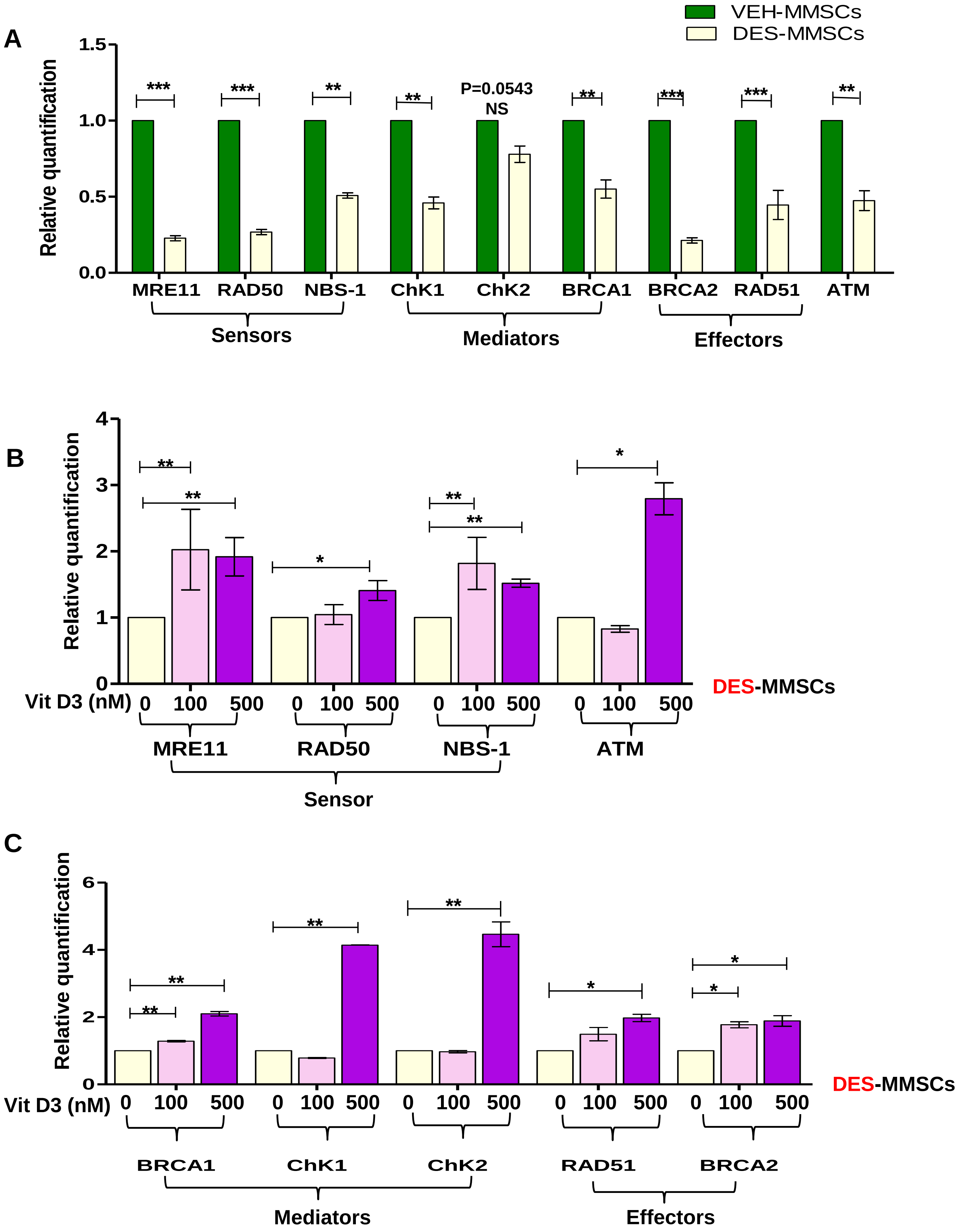

Supplemental Fig. 2

Supplemental Figure 2. The effect of VitD3 on the RNA expression of DNA repair genes in DESMMSCs (A) Baseline RNA expression of several DNA DSB repair-related genes, including MRE11, RAD50, NBS-1, BRCA1, BRCA2, RAD51, CHK1, CHK2 and ATM was examined by q-PCR in DESMMSCs and VEH-MMSCs. (B) The RNA expression of DNA DSB repair-related genes, including MRE11, RAD50, and NBS-1 as well as ATM was determined by q-PCR in response to VitD3 in DESMMSCs. (C) The mRNA expression of the DNA repair mediator gene (BRCA1, ChK1, and Chk2) and DNA DSB repair effector genes (BRCA2 and RAD51) was measured by q-PCR in the presence and absence of Vitamin D3. The mRNA levels were normalized to 18S rRNA. Normalized values were used to generate the graph. Data are presented as mean± SEM of triplicate measurements with $^{*}P<0.05$, $^{**}P<0.01$, $^{***}p<0.001$.

Supplemental Figure 3. In vitro study design (A) untreated DES-MMSCs, (B) DES-MMSCs were treated with mirin (20 µg/ml) for 9 hrs, (C) DES-MMSCs were treated with camptothecin (CPT) treatment (20 µg/ml) for 3 hrs. (D) DES-MMSCs were treated with mirin (20 µg/ml) for 9 hrs followed by CPT treatment (20 µg/ml) for 3 hrs, (E) DES-MMSCs were pretreated with VitD3 (100nM) for 1hr, then treated with mirin (20 µg/ml) for 9 hrs, followed by camptothecin (20 µg/ml) for 3 hrs. (F) DES-MMSCs were treated with mirin (20 µg/ml) in combination with VitD3 (100 nM) for 9 hrs. The cells were washed and treated with camptothecin (20 µg/ml) for 3 hrs. (G) DES-MMSCs were treated with mirin (20 µg/ml) for 9 hrs, then treated with camptothecin (20 µg/ml) for 3 hrs. VitD3 (100nM) was added for the entire12-hr treatment with mirin and camptothecin, respectively. These group labellings were used for figures 5 and 6 in the original manuscript.

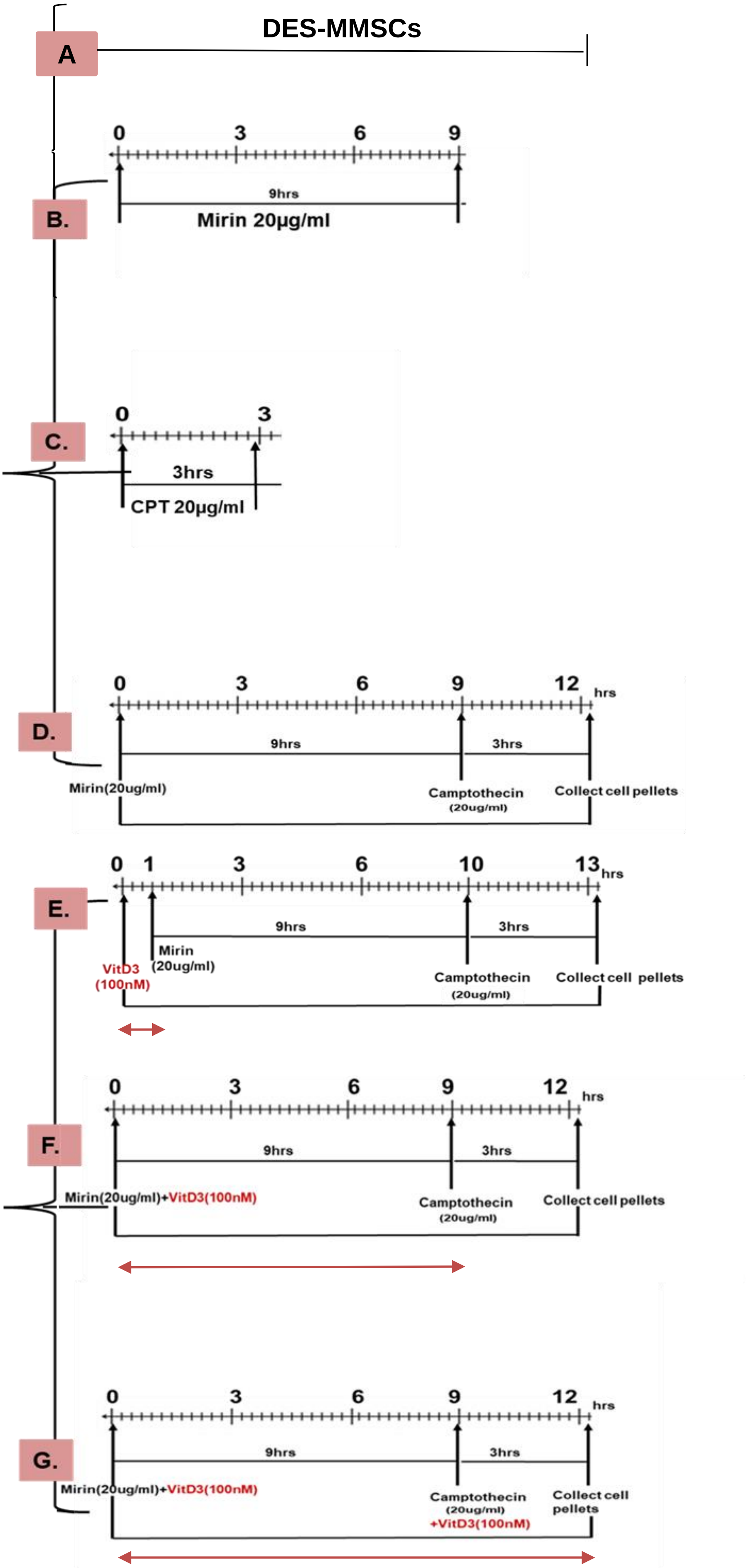

Supplemental Fig. 3

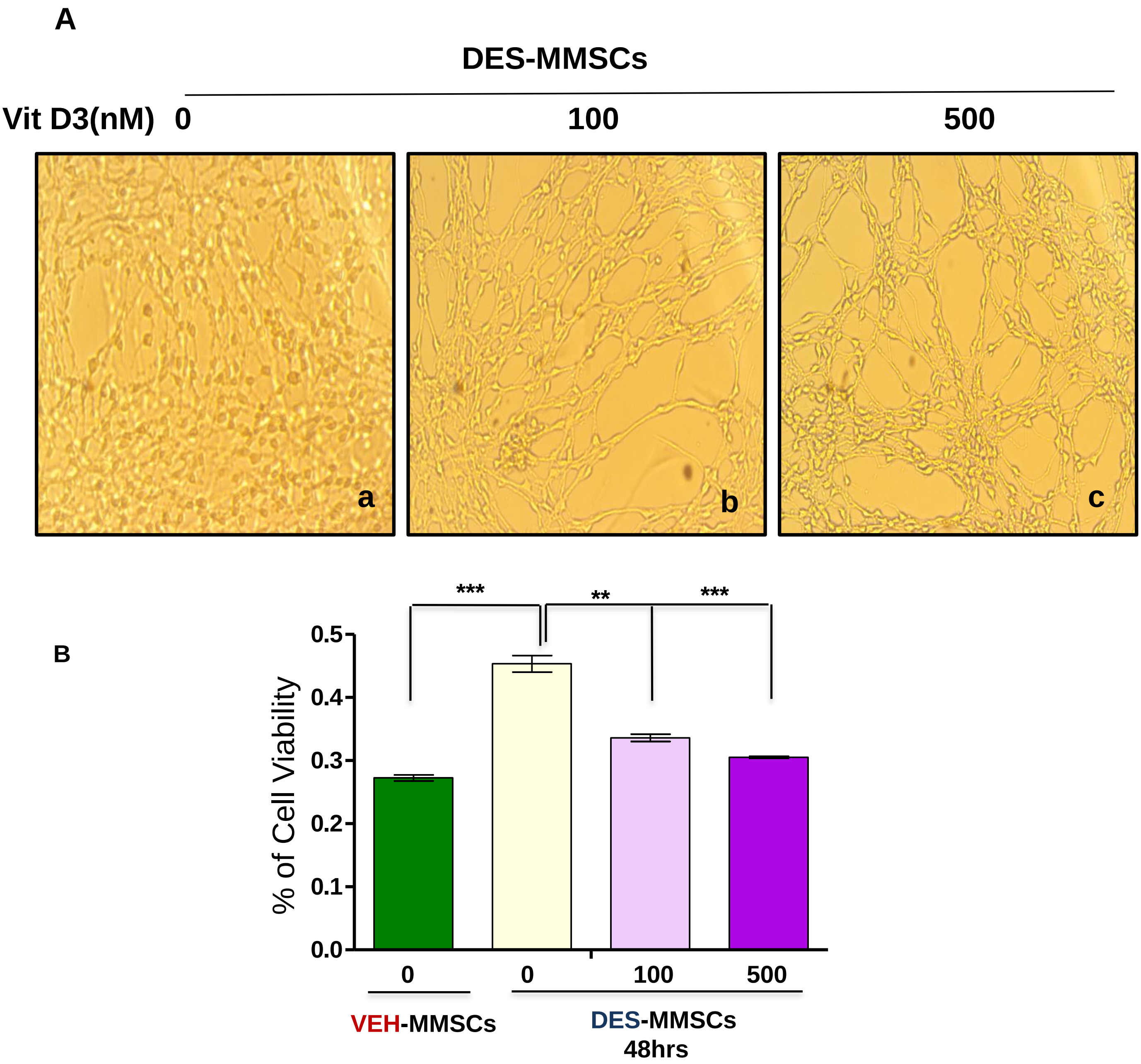

Supplemental Figure 4. The effect of VitD3 on DES-MMSCs proliferation (A) Phase-contrast images: (a) DES-MMSCs, (b) DES-MMSCs treated with 100 nM VitD3, and (c) DES-MMSCs treated with 500 nM VitD3. Scale bar = 100 µm. (B) MTT assay showing the percentage of viable cells in untreated VEH-MMSCs and DESMMSCs without and with VitD3 (at 100 nM and 500 nM) treatment. Experiments were performed in triplicate, n= 5 biological replicates. All graphs show mean ± SEM. Two-tailed unpaired Student's t-test was used for the statistical analysis.
